# Aminoglycoside ototoxicity and hair cell ablation in the adult gerbil: A simple model to study hair cell loss and regeneration

**DOI:** 10.1016/j.heares.2015.03.002

**Published:** 2015-07

**Authors:** Leila Abbas, Marcelo N. Rivolta

**Affiliations:** Centre for Stem Cell Biology and Department of Biomedical Sciences, University of Sheffield, Sheffield S10 2TN, United Kingdom

**Keywords:** SGN, Spiral Ganglion Neuron, ABR, Auditory Brainstem Response, PFA, Paraformaldehyde, EDTA, Ethylenediaminetetraacetic acid, PBS, Phosphate Buffered Saline, BSA, Bovine Serum Albumin, DAPI, 4,6-diamidino-2-phenylindole, MSBB, Methyl salicylate and Benzyl benzoate, ANOVA, Analysis of variance, RWM, Round window membrane, OHC, Outer hair cells, IHC, Inner hair cells, MBP, Myelin basic protein

## Abstract

The Mongolian gerbil, *Meriones unguiculatus*, has been widely employed as a model for studies of the inner ear. In spite of its established use for auditory research, no robust protocols to induce ototoxic hair cell damage have been developed for this species. In this paper, we demonstrate the development of an aminoglycoside-induced model of hair cell loss, using kanamycin potentiated by the loop diuretic furosemide. Interestingly, we show that the gerbil is relatively insensitive to gentamicin compared to kanamycin, and that bumetanide is ineffective in potentiating the ototoxicity of the drug.

We also examine the pathology of the spiral ganglion after chronic, long-term hair cell damage. Remarkably, there is little or no neuronal loss following the ototoxic insult, even at 8 months post-damage. This is similar to the situation often seen in the human, where functioning neurons can persist even decades after hair cell loss, contrasting with the rapid, secondary degeneration found in rats, mice and other small mammals. We propose that the combination of these factors makes the gerbil a good model for ototoxic damage by induced hair cell loss.

## Highlights

• We have explored different paradigms of ototoxicity in the gerbil model.
• The gerbil sensory hair cells seem relatively resiliant to gentamicin.
• Hair cell damage is not followed by significant loss of spiral ganglion neurons.
• This generates a simple model for hair cell loss that resembles human lesions.
• This model should be ideal to study regenerative strategies and cochlear implantation.

## 1 Introduction

Since their introduction in the 1940s, aminoglycoside antibiotics have been recognised clinically for their off-target effects of ototoxicity. When used in combination therapy with a loop diuretic such as ethacrynic acid, the often-reversible deafness seen with the antibiotic alone was rapidly induced and permanent (Brown et al., 1974; Mathog et al., 1969). Such damage was found to be caused by the death of the sensory cells of the specialised hearing epithelium, the organ of Corti, located within the bony shell of the cochlea. The destruction of the three rows of outer hair cells and single row of inner hair cells would eventually lead to loss of the surrounding supporting cells and the replacement of the organ with a flattened epithelium of scar tissue, and accompanying profound deafness in the patient. However, as the field of regenerative medicine moves forward, this damaged epithelium becomes a potential target for therapeutic intervention, whether it be the idea of recreating the organ of Corti, or in its role as a model for cochlear implantation studies.

A sequela to the death of the organ of Corti is often the secondary loss of the spiral ganglion neurons (SGNs) which innervate the hair cells. This loss occurs with varying rapidity in different species. For instance in the guinea pig, a substantial abrogation of SGNs is observed 7 days after aminoglycoside treatment (Kong et al., 2010), whereas in human patients, remaining SGNs have been found several decades after hair cell loss is thought to have occurred (Ghorayer et al., 1980).

The gerbil is a well-established model for auditory research given its particular hearing physiology (Otto and Jürgen, 2012). On account of its ethology in the wild, the animal has a broad frequency range of hearing – low frequencies are used when ‘drumming’ with the hind limbs as a warning communication; at the other end of the auditory spectrum, animals ‘chirp’ at each other up to a level of around 25 kHz. This overlap with the human hearing range arguably makes the gerbil a more relevant model for hearing loss than high-frequency specialists such as the mouse or rat. Moreover, the species is surgically robust, with the relatively large cochlea easily accessed through the thin bone of the auditory bulla making it particular well suited for experiments exploring therapeutic strategies requiring cell or drug delivery.

Remarkably though, while reliable protocols have been developed for the neuropathic damage of the spiral ganglion (Lang et al., 2005; Schmiedt et al., 2002), a simple and robust method to induce ototoxic lesions of the hair cells is not available for this species. Current protocols involve the topical application of aminoglycosides using slow-releasing gels or repeated application of aminoglycosides by transtympanic injections (Polgar et al., 2001; Wanamaker et al., 1999). Both methods are invasive and, at least in our hands, have proven unreliable.

Here we present data showing that the gerbil can be used as a model for rapid and permanent aminoglycoside-induced hearing loss using a ‘one-shot’ protocol, in which a single dose of kanamycin is accompanied by a dose of the loop diuretic furosemide. This is a refinement of experiments carried out in other species, where repeated, often toxic, dosage regimes have been employed.

## 2 Materials and methods

### 2.1 Animals

Mongolian gerbils from an in-house breeding colony (originally sourced from Charles River, Germany) were raised and aged between 3 and 6 months at the beginning of the protocol – no effect of age was noted on the initial hearing threshold measured. A mixture of males and females were used – no difference between sexes in hearing ability has been observed in our colony. All experiments were carried out in accordance with the Sheffield University Ethical Review Committee and under a Home Office Project Licence authority, conforming to UK and EU legislation.

### 2.2 Ototoxicity models

For systemic assays, animals were injected sub-cutaneously with a solution of gentamicin sulphate or kanamycin sulphate in normal saline (400–500 mg/kg; Sigma, Gillingham, U.K.) followed 20–30 min later by an intra-peritoneal injection of bumetanide (50 mg/kg; Sigma) or furosemide solution (100 mg/kg; Sigma). For topical application to the cochlea, anaesthesia was induced with isoflurane, the auditory bulla was accessed through a retro-auricular incision and windowed using a scalpel blade. Aminoglycoside and diuretic solutions (as above) were applied directly to the round window; in some cases, animals were implanted with a gelatin sponge (Cutanplast, Sheffield, U.K.) pre-soaked in an aminoglycoside/diuretic mixture. The bulla was sealed using a plug of fascia held in place with Vetbond adhesive (3 M, Bracknell, U.K.). The muscle layer and wound were closed with absorbable Vicryl suture material (Ethicon, Norderstedt, Germany) and the animal allowed to recover.

### 2.3 Auditory measurements

Animals were sedated with a ketamine/xylazine mix and placed on a heat mat to maintain body temperature. The System 3 digital signal processing package of hardware and software (Tucker Davies Technologies (TDT), Florida) was used to present click stimuli ranging from 110 dB to 20 dB at a rate of 20 s^−1^ in decreasing 10 dB steps. Tone stimuli were presented as pure tone pips lasting 5ms at 80 dB, at frequencies ranging from 6 kHz to 38 kHz in 4 kHz steps or at 2 kHz, 4 kHz, 8 kHz, 16 kHZ and 32 kHz at an intensity ranging from 20 dB to 90 dB. The sounds were presented to the animal via a ‘closed field’ method – a 3 mm diameter, 10 cm tube leading from the speaker was inserted into the auditory meatus until its tip was close to the tympanic membrane. Auditory brainstem response (ABR) measurements were recorded using 27G subdermal needle electrodes placed at the vertex of the skull (recording electrode) and the ipsilateral mastoid process (reference electrode), with a ground electrode placed dorsally adjacent to the tail. The differential voltage induced by the presentation of the sound stimulus was thus recorded. Each stimulus was presented 500 times, thus the wave generated represented an average response over this time. For each intensity, the amplitude of the response between the wave ii positive peak (P2) and the wave iii negative peak (N3), representing the neural activity within the cochlear nucleus and the superior olivary complex respectively, was measured, giving a ‘read-out’ of activity within the central auditory pathway (Burkard et al., 1993; Boettcher et al., 2006). The click ABR threshold was calculated as the sound level required to produce a voltage response two standard deviations above the mean noise level for each recording (May et al., 2002). All experiments were carried out in a sound-proof, custom-made audiology booth (AGS Noise Control Ltd, Melton Mowbray, U.K.).

### 2.4 Immunohistochemistry

Temporal bones were fixed by immersion in 4% paraformaldehyde (PFA; pH 7.4) for 24–48 h at 4 °C and decalcified in 0.5 M ethylenediaminetetraacetic acid (EDTA) pH8 for a week at 4 °C. Samples were taken through an ascending sucrose/PBS series (7.5%, 15%, 22.5%, 30; several hours/overnight in each stage). Following embedding in Cryo-M-Bed medium (VWR, Lutterworth, U.K.), 12 μm sections were taken on a Bright cryostat onto gelatine/chrome alum-coated glass slides. The tissue sections were defrosted, rehydrated and briefly re-fixed with 4% PFA, rinsed with 0.1% Triton/PBS and blocked in 5% donkey serum/1% B.S.A. before incubation overnight at 4 °C with the following antibodies: anti-αTubulin (1:150, mouse monoclonal; Sigma), anti-βIII–Tubulin (1:150, mouse monoclonal; Sigma), anti-espin (1:100, rabbit polyclonal; Sigma), anti-myelin basic protein (MBP; 1:75, mouse monoclonal; Millipore), anti-MyoVIIA (1:100, rabbit polyclonal; kind gift, C. Petit), anti-OCP2 (1:100, rabbit polyclonal, kind gift R. Thalmann), anti-Neurofilament 200 (NF-200; 1:200, rabbit polyclonal; Sigma), anti-Na, K-ATPase α3 subunit (NKAα3; Santa Cruz, Insight Biotechnology, Wembley, U.K.), anti-CtBP2 (BD Biosciences, Oxford, U.K.). Detection was carried out by incubating for 2 h at room temperature with anti-mouse Alexa 488 and anti-rabbit Alexa 568 (Life Technologies, Paisley, U.K.) and the tissue counter-stained with 4,6-diamidino-2-phenylindole (DAPI) (Sigma) before mounting in Vectashield (Vector Laboratories, Peterborough, U.K.). Images were taken on a Zeiss Axiophot microscope using Axiovision software and figures assembled using Adobe Photoshop 7.0. Whole-mount staining of intact cochleae was carried out essentially as described in MacDonald and Rubel (2008). Briefly, fixed cochleae were decalcified as above, rinsed in PBS and incubated with primary antibodies (anti-βIII–Tubulin and anti-MYOVIIA as above) for several days with agitation at 4 °C. After washing, samples were incubated at 4 °C with anti-mouse Alexa 488 and anti-rabbit Alexa 568 for 3–5 days. After washing, samples were dehydrated through an increasing ethanol gradient and cleared through MSBB, in a 5:3 ratio of methyl salicylate and benzyl benzoate (Sigma), for 2 days. Imaging was then carried out using a Zeiss LSM510 inverted confocal microscope. Images were analysed using FIJI (http://fiji.sc/Fiji) and figures assembled in Photoshop as above.

### 2.5 Phalloidin staining

Temporal bones were fixed and decalcified as described above. Cochlear epithelia were dissected out and washed in 0.1% Triton/PBS prior to incubation overnight in Alexa Fluor 568 phalloidin (2 units/ml; Life Technologies). Following copious washes in 0.1% Triton/PBS, samples were mounted in Vectashield and imaged as above.

### 2.6 Spiral ganglion density calculation

SGNs in Rosenthal's canal were stained for βIII–Tubulin and counted from images taken on a Zeiss Axiophot microscope as before. Apical, mid and basal turns were counted from a minimum of five, mid-modiolar sections per cochlea. Sections counted were at least 36 μm apart to prevent over-counting. Cell density was calculated by measuring the area of Rosenthal's canal using FIJI (http://fiji.sc/Fiji) and calculating the number of cells per mm^2^. One-way ANOVA and unpaired t-tests for significance were carried out in Prism (Graphpad Software, San Diego, U.S.A.).

## 3 Results

### 3.1 Topical gentamicin application does not induce obvious cochlear hair cell loss

**Fig. 1:**
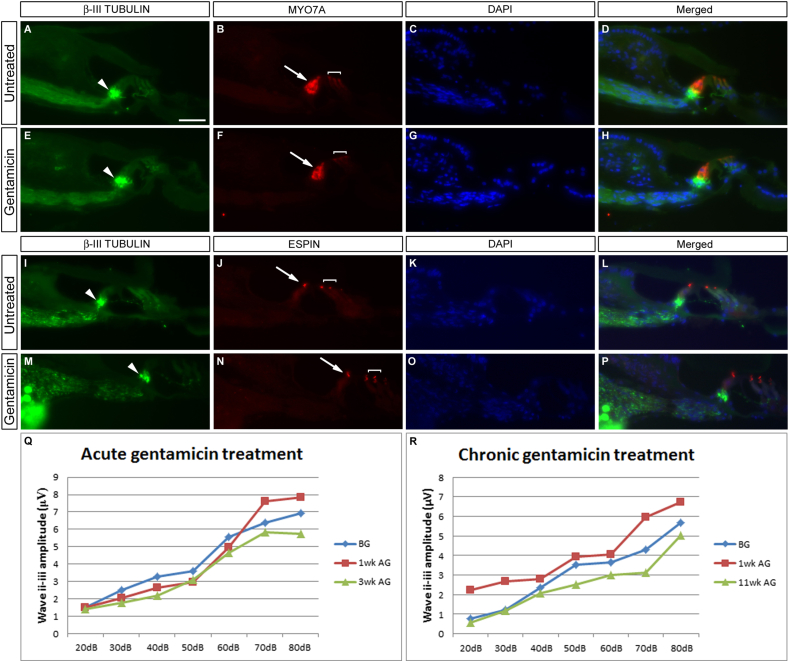
Topical application of gentamicin has no effect on the hearing organ. Gentamicin sulphate solution was applied directly to the round window membrane either acutely (E–H, Q) or as in a ‘slow release’ paradigm on a gelatine sponge (M–P, R). Immunofluorescence for β-III TUBULIN and the hair cell markers MYO7A (B, D, F, H) and ESPIN (J, L, N, P) show that there is no effect on the organ of Corti in either situation – comparison with untreated contralateral ears (A–D; I–L) shows no overt difference in structure or integrity of the inner and outer hair cells. Sections are counterstained with DAPI to delineate cell nuclei. There is no disruption to the innervation of the IHCs by spiral ganglion neurons (arrowheads, A, E, I, M). Click ABR measurements were taken at intervals post-treatment – no difference in response was found down to 20 dB with either treatment regime (Q, R). Arrows point to inner hair cells, brackets delineate the three rows of outer hair cells. Scale bar – 50 μm.

Our first investigations focussed on topical application of the aminoglycoside to the cochlea. The auditory bulla was fenestrated and 400 μg of gentamicin sulphate in aqueous solution was applied to the round window membrane (RWM), either directly (n = 3); or on a pre-soaked piece of gelatin sponge to effect a ‘slow release’ (n = 1). However, contrary to results seen in the guinea pig (Yang et al., 2010) and previously in the gerbil (Sheppard et al., 2004) there was no obvious loss of hair cells (Fig. 1). Immunofluorescence was performed on sectioned cochlear tissue – the markers for IHCs and OHCs, MYO7A and ESPIN showed normal expression in treated vs. untreated contralateral ears throughout the entire extent of the cochlea (Fig. 1A–D vs. E–H and I–L vs. M–P), suggesting that no damage had occurred on account of this treatment. Representative sections from the basal (A–H) and mid (I–P) turns are shown in Fig. 1; similar results were observed at all three axial levels for both treatment paradigms. Innervation of the organ of Corti was assayed with the neural marker β-III TUBULIN (Hallworth and Luduena, 2000) and no apparent loss of innervation to the inner hair cells was noted after gentamicin treatment (see white arrowheads, Fig. 1). These results were confirmed at the functional level (Fig. 1Q, R). ABR click thresholds stayed at 20 dB (data not shown), showing no shift above the measured starting threshold and similar click response profiles were observed up to 80 dB, with animals being tested at regular intervals post-treatment. In the acute case shown (Fig. 1Q), the procedure was terminated at 3 weeks post-application, whereas the chronic application was allowed to run for 11 weeks lest there be a slow degeneration – however, this was clearly not the case, with the measured response amplitudes remaining consistent. No behavioural phenotype indicative of vestibular toxicity, such as circling or head bobbing, was noted in these animals. These results, although involving a small cohort of animals, suggested to us that the use of gentamicin would not be an appropriate choice as an efficient ototoxic agent in this system and consequently gentamicin was not pursued any further.

### 3.2 Combined application of kanamycin and bumetanide induces only mild damage

**Fig. 2:**
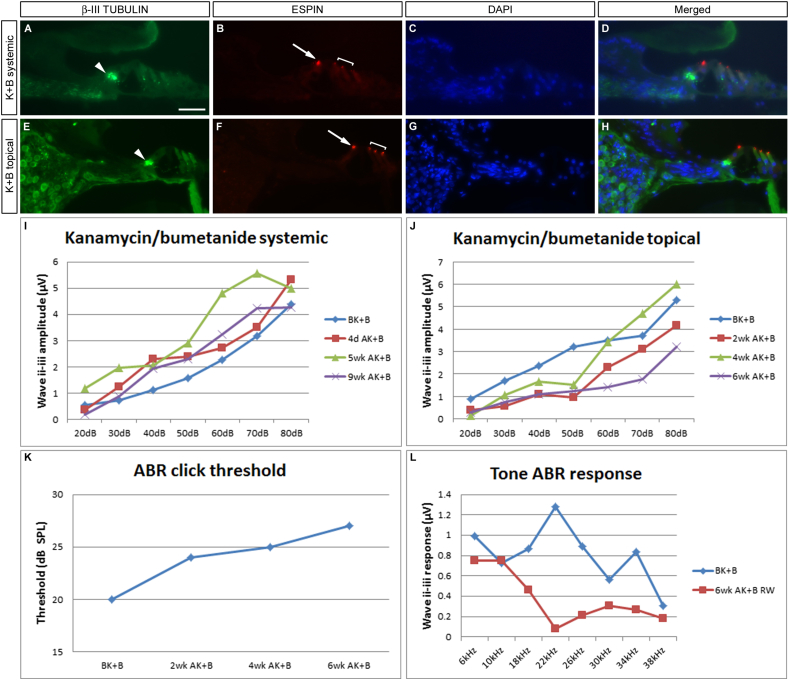
Application of kanamycin and bumetanide has minor effects on hearing. A combinatorial approach using kanamycin and bumetanide has no effect when dosed systemically (A–D, I), and only gives a moderate loss of hearing when given as a topical, chronic application (E–H, J–L). Immunofluorescence for ESPIN shows that in both cases the organ of Corti is intact (arrows and brackets B, F, compare with untreated control, Fig. 1J, L) and β-III TUBULIN staining shows that the innervation to the hair cells is normal (arrowheads, A, E). Sections are counterstained with DAPI to delineate cell nuclei. Typical sections from each sample are shown – apical turn (A–D), basal turn (E–H). Click ABR responses are maintained at 20 dB in the systemic treatment (I). There is a minor increase in auditory threshold in the topical case 6wk after treatment (J, K), and this functional decrement is most pronounced in the mid-frequency range, from 18 to 30 kHz (L). Arrows point to inner hair cells, brackets delineate the three rows of outer hair cells. Scale bar – 50 μm.

Kanamycin has also been widely used as a means of inducing cochlear ototoxicity in other animal models, such as the rat (Astbury and Read, 1982) or the guinea pig (Aran et al., 1975; Poirrier et al., 2010). However, studies have demonstrated that its effects are strongly potentiated when used in conjunction with a loop diuretic, such as ethacrynic acid (Brummett, 1981) or bumetanide (Taylor et al., 2008). In a pilot experiment, both drugs were administered in a systemic treatment paradigm with a single dose of kanamycin followed forty minutes later by a dose of bumetanide, which clearly induced diuresis (n = 1). Alternatively, drugs were applied together on gelatine sponge topically to the RWM as above (Section 3.1) (n = 1). Again however, neither regime generated an obvious loss of cochlear hair cells as shown by the normal expression of ESPIN and MYO7A in the inner and outer hair cells throughout the cochlea. Representative turns are shown in Fig. 2A–H. Systemic treatment did not result in a loss of hearing (Fig. 2I) – a robust response to a 20 dB click was preserved for the nine week duration of the protocol. However, a slight decrement in hearing was observed in the topical, gelatine sponge treatment paradigm (Fig. 2J–L). The auditory threshold was raised from 20 dB to 27 dB (Fig. 2K) over the course of the experiment and an analysis of the responses to pure tone frequencies suggests that this loss occurs predominantly in the mid-frequency range, with 18–34 kHz being most affected (Fig. 2L). Albeit significant, this loss is too subtle for our purposes of inducing rapid and substantial hearing loss and again, this dosing regimen was not pursued further.

### 3.3 Treating with kanamycin and furosemide gives substantial hair cell loss

**Fig. S1:**
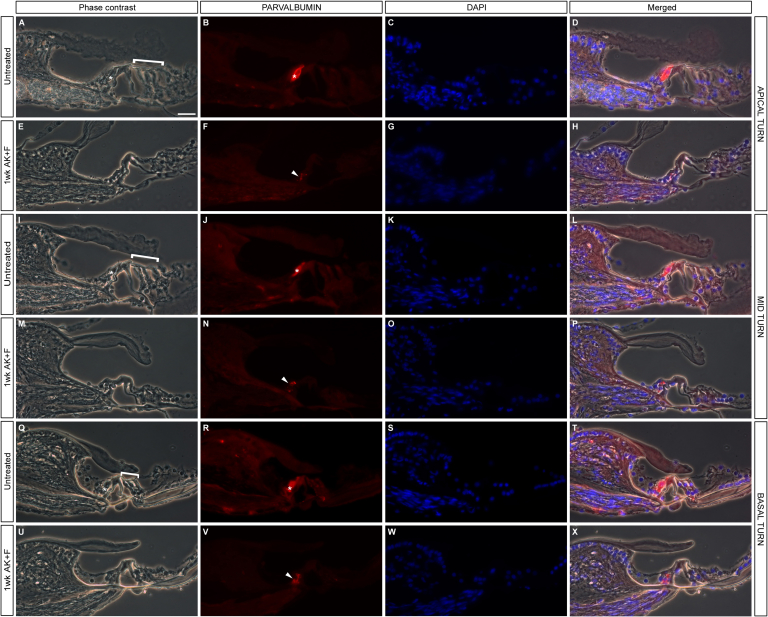
Phase contrast imaging demonstrates the loss of the hair cells in kanamycin/furosemide treated animals. Untreated (A–D, I–L, Q–T) and kanamycin/furosemide treated animals at one week (E–H; M–P; U–X) were stained for the presence of the calcium binding protein PARVALBUMIN, which marks the inner hair cells in intact animals (asterisks, B, J, R), and the sensory afferents (see Fig. S2 for further demonstration of this); whereas only the nerve fibres are labelled in the treated samples (arrowheads, F, N, V). Phase microscopy imaging demonstrates the loss of inner (asterisks, A, I Q) and outer hair cells (brackets, A, I Q). Representative apical (A–H), mid (M–P) and basal (Q–X) sections are shown, and are counterstained with DAPI (G, K, O, S, W). Scale bar – 50 μm throughout.

**Fig. 3:**
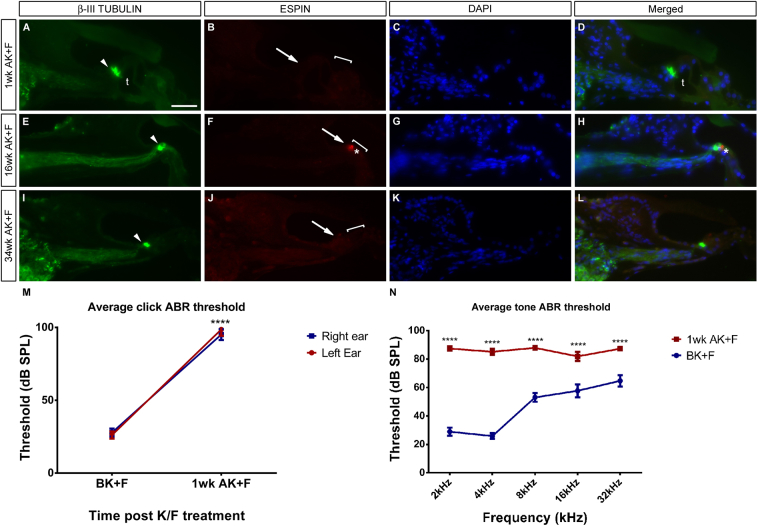
Systemic application of kanamycin and furosemide destroys inner and outer hair cells. Systemic application of kanamycin and furosemide gives a rapid and comprehensive destruction of both inner and outer hair cells. Immunofluorescence for the hair bundle marker ESPIN shows a complete loss of immunoreactivity 1wk after drug administration, implying a loss of hair cell function (compare arrows and brackets in B with the control situation in Fig. 1J). The tunnel of Corti is present at this stage (t, A, D). The hair bundle loss is permanent – at both 16wk and 34wk post-treatment, there is no restoration of the hair bundle (arrows and brackets, F, J) and the organ of Corti has degenerated – asterisks in F, H point to a small area of anomalous espin expression which would not constitute a functioning hair cell and may represent a hair cell in the process of being engulfed and destroyed. However, the presence of the spiral ganglion neuron processes remains intact (arrowheads, A, E, I.) DAPI is used as a counterstain as previously. Average click ABR thresholds rise considerably after treatment (M), from around 27 dB initially to around 97 dB after seven days. No difference is seen between the responses of the left and right ears (left ear n = 9; ****p < 0.0001; right ear n = 15; ****p < 0.0001). Pure tone ABR threshold measurements one week after kanamycin/furosemide treatment demonstrate an equivalent loss of response across the frequency range measured, 2–32 kHz (N) (n = 6; ****p < 0.0001). Mean and SEM plotted throughout. Scale bar – 50 μm.

The loop diuretic furosemide has also been widely used to strengthen the ototoxic effects of aminoglycosides – for examples, see (Alam et al., 1998; Brummett et al., 1975; Hirose et al., 2011) and references therein. In gerbils treated with a single dose of kanamycin only, no evidence of hair cell loss is observed (data not shown). However, when a single, systemic kanamycin dose is followed shortly afterwards with an injection of furosemide, there follows a rapid and substantial loss of both inner and outer hair cells (Fig. 3). Immunofluorescence for the protein ESPIN shows a complete loss of immunoreactivity one week after drug administration (Fig. 3A–D), implying a loss of hair cell function – when compared with untreated controls, there is an absence of staining in the single row of inner hair cells and all three rows of outer hair cells. Equivalent results are found when samples are stained for the presence of MYO7A (data not shown). Phase contrast imaging coupled with immunofluorescence for the calcium binding protein PARVALBUMIN at one week post-treatment confirms these results (Fig. S1), such that the PARVALBUMIN-positive inner hair cells are lost at all levels of the cochlea, leaving behind PARVALBUMIN-positive afferent fibres projecting into the damaged organ of Corti. Meanwhile, however, the tunnel of Corti is still present at this stage and the architecture of the hearing organ appears to be relatively intact (‘t’; Fig. 3A, D). As would be expected from work in other mammalian species though, this hair bundle loss is permanent. At later time points, for example at 16wk and 34wk post-treatment, there is little to no immunoreactivity for espin and the organ of Corti has degenerated, with a collapse of the tunnel of Corti and a flattening of the putative remaining support cells (Fig. 3E–L). However, the presence of spiral ganglion neurons afferent processes remains intact throughout (white arrowheads, A, E, I), even eight months after the acute ototoxic insult and in spite of the hair cell loss (see Section 3.4 for further investigation of this).

The auditory brainstem response (ABR) was measured in fifteen animals both prior to and at intervals subsequent to the ototoxic challenge (Fig. 3M, N). As alluded to in Section 3.1, the ABR evoked by a broad-spectrum click ranging from 20 dB to 110 dB was recorded before the drug dosing, one week later (Fig. 3M) and at frequent intervals until the end of the procedure (data not shown). Auditory thresholds were raised on average by almost 69 dB after one week (n = 15, 24 ears; Fig. 3M) with rises in individual animals ranging from 36 to 97 dB. At the end of the procedure there was an average increase in the auditory threshold of 64 dB (n = 4; data not shown). This is not significantly different to the loss seen after one week, with average measured thresholds being 94 dB and 93 dB respectively implying that the majority of the damage seen occurs as an acute response to the ototoxic insult. There was no difference in the response between the left and right ears, with the average threshold increase at one week post-insult being of 72 dB and 67 dB respectively (Fig. 3M).

The responses to individual frequency pips from 2 kHz to 32 kHz at an intensity range of 20 dB–90 dB were also measured (n = 6 animals; 12 ears measured; Fig. 3N). A highly significant rise in threshold was observed across the frequency spectrum measured (see Table 1), implying that the hair cells were equally sensitive to the treatment along the length of the cochlea.

### 3.4 Hair cell counts one week after kanamycin/furosemide treatment

**Fig. 4:**
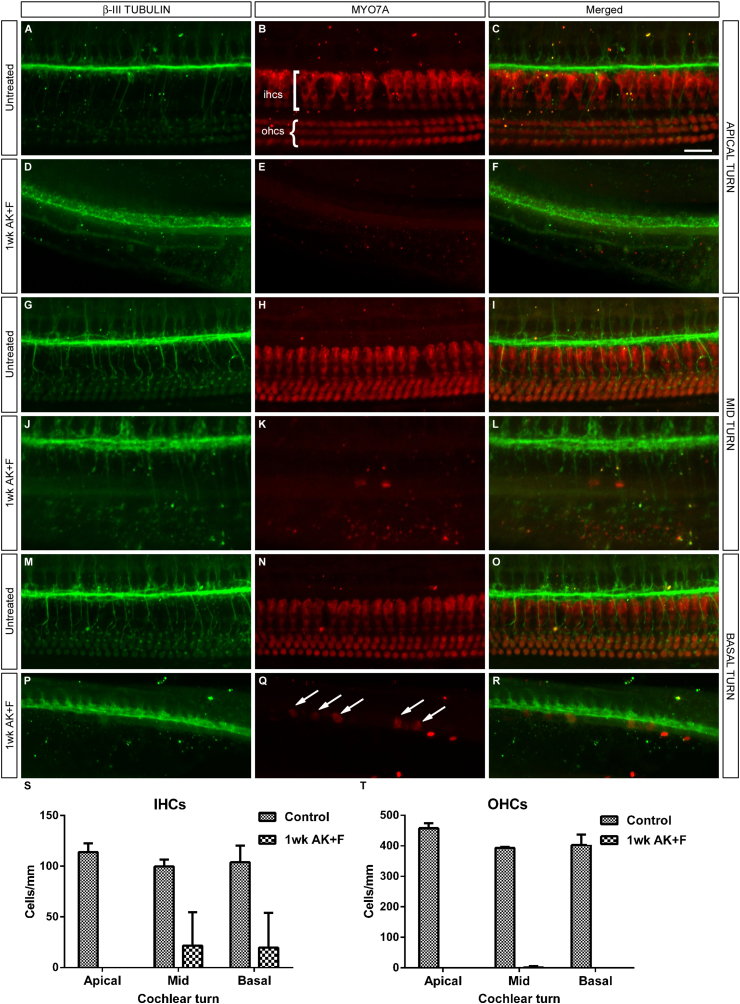
Quantification of hair cell loss at all three cochlear levels. Whole-mount immunostaining of the cochlea with antibodies directed towards βIII-TUBULIN as a marker for spiral ganglion neurons (A, D, G, J, M, P and merged panels C, F, I, L, O, R) and MYO7A (B, E, H, K, N, Q and merged panels) to show the hair cells demonstrates a marked and quantifiable loss of both IHCs and OHCs at one week after kanamycin/furosemide treatment (D–F, J–L, P–R) when compared with untreated controls (A–C, G–I, M–O). Apical (A–F) mid (G–L) and basal (M–R) turns have an equivalent loss of OHCs, whereas there is a handful of IHCs remaining basally (arrows, Q) – however, these cells have lost their elongated morphology and are likely to represent a dying population. Cell counts were carried out and the mean number of each cell type per turn is displayed graphically in S, T; error bars represent SEM Scale bar – 25 μm throughout A–R.

The damage to both inner and outer hair cells is substantial. To quantify the degree of hair cell loss observed, a cohort of animals was treated with kanamycin and furosemide and the cochleae were examined one week later using whole-mount immunofluorescence (MacDonald et al., 2008), a technique which allows the individual turns of the cochlea to be imaged in an ‘in situ’ context (Fig. 4). Staining for MYO7A immunoreactivity and subsequent cell counting analysis (Table 2) showed a massive loss of both inner and outer hair cells, demonstrated by the loss of MYO7A staining, at all levels of the cochlea (Fig. 4B, E, H, K, N, Q). A handful of IHCs remain in the basal turn of the treated condition (Fig. 4Q), but these are clearly dysmorphic compared to their untreated counterparts (Fig. 4N) and may represent a dying population yet to be cleared by macrophages. The position of the organ of Corti was determined by the position of the underlying β-III TUBULIN staining (Fig. 4A, D, G, J, M, P).

### 3.5 Non-sensory cells retain their identity

**Fig. 5:**
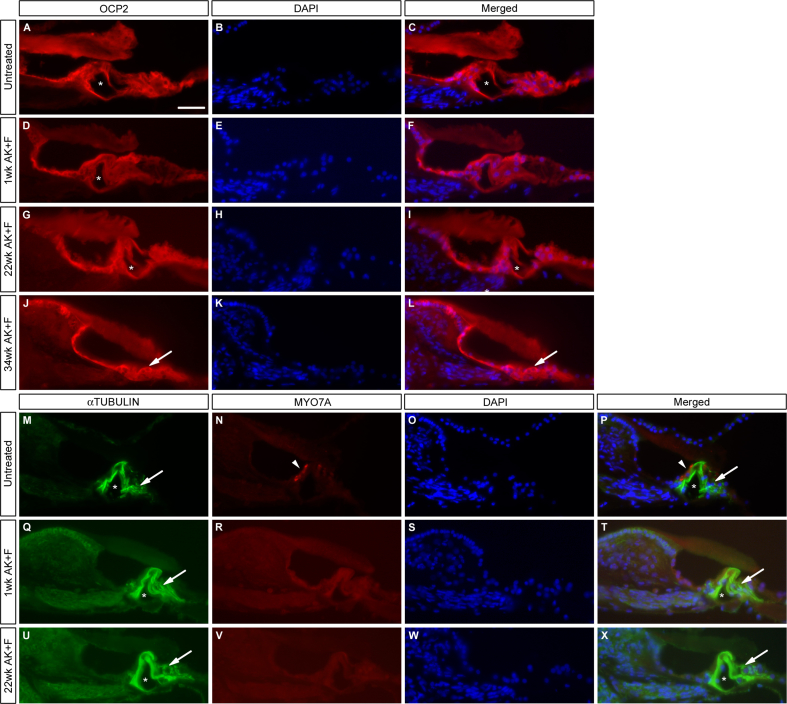
Expression of the markers OCP2 and αTUBULIN in drug treated animals. The proteins OCP2 and acetylated αTUBULIN are markers for non-sensory supporting cells in the organ of Corti. Their expression remains high in the cochlea at all stages post-treatment with kanamycin/furosemide. OCP2 levels are comparable with the untreated condition (A–C) at 1wk (D–F), 22wk (G–I) and 34wk (J–L), suggesting that the damaged epithelium retains some level of differentiation, although it has become flattened in appearance by 34wk (J–L). Asterisks in A, C, D, F, G, I mark the intact tunnel of Corti; arrows in J, L indicate where this structure has collapsed. Costaining of MYO7A with αTUBULIN (M–X) shows a similar profile. In the untreated condition (M–P), the inner hair cells are clearly visible (arrowheads, N, P) – this staining is lost at all stages post-treatment as before (Q–X). The support cells, marked by αTUBULIN, remain intact and differentiated (arrows, M, P, Q, T, U, X) and the tunnel of Corti remains open (Q–X) up to 22wk post-treatment (asterisks, M, P, Q, T, U, X). DAPI is used as a nuclear counter-stain. Scale bar – 50 μm throughout.

There have been reports in the literature of the loss of support cell identity as a secondary consequence of the loss of the sensory hair cells – the support cells become flattened and assume a cuboidal, epithelial appearance (Hellier et al., 2002; Hirose et al., 2011; Huizing et al., 1987; Taylor et al., 2012). To examine this in the kanamycin/furosemide treated gerbil, sections of the cochlea taken at the various time points were examined for their expression of OCP2, a component of the SCF ubiquitin ligase complex (Henzl et al., 2004), which is abundant in the non-sensory cochlea cells, from the interdental cells of the spiral limbus to the root cells of the spiral ligament; and acetylated αTUBULIN, a marker for Deiter's cells and inner and outer pillar cells (Saha et al., 2000; Tannenbaum et al., 1997). Expression levels of OCP2 remained comparable to the untreated condition at all stages post-insult, from 1 week to 34weeks (Fig. 5A–L). Similarly, levels of acetylated αTUBULIN remained equivalent in untreated vs. treated animals (Fig. 5M–X), in the pillar cells and Deiter's cells. Although we have not performed a quantitative analysis of the expression of these markers to categorically confirm they remained unchanged, their qualitative visualization would suggest that the support cells are capable of surviving for long periods in the absence of hair cells in the gerbil. The tunnel of Corti remained open and intact for a considerable time post-damage (16wk, data not shown; 22wk Fig. 5G–I, asterisks) and classical support cell morphology was maintained, similar to that seen when compared with untreated (Fig. 5A–C) and early lesioned animals (1wk, Fig. 5D–F). However, after long-term damage, a change in cell morphology was in evidence – samples at 34wk displayed a collapse of the tunnel of Corti (arrows, Fig. 5J–L) and a clear flattening of the pillar cells.

### 3.6 Peripheral neural processes into the damaged organ of Corti remain intact

**Fig. S2:**
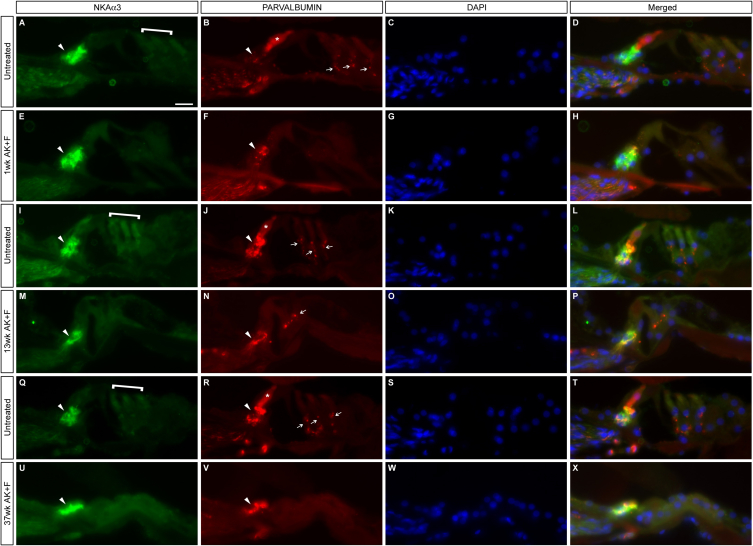
Sensory afferents do not degenerate in the absence of inner hair cells. Untreated (A–D, I–L, Q–T) and kanamycin/furosemide treated animals at different time points (1wk, E–H; 13wk M–P; 37wk U–X) were co-stained for the presence of the sensory afferent marker Na, K-ATPase α3 subunit (NKAα3) and the calcium-binding protein PARVALBUMIN (as per Fig. S1). The latter marks both the sensory afferents and the inner hair cells in intact animals (arrowheads and asterisks, B, J, R), whereas only the nerve fibres are labelled in the treated samples (arrowheads, F, N, V). Colabelling with the marker NKAα3 (arrowheads, A, E, I, M, Q, U) again demonstrates the presence of fibres within the damaged organ of Corti, even at 37wk post-treatment. Similar results were seen at apical (A–H), mid (Q–X) and basal (I–Q) levels of the cochlea. There is also innervation of the OHCs (square brackets, A, I, Q) by PARVALBUMIN positive fibres (arrows, B, J, R) – this staining is either lost (F, V) or degenerative (N) in treated animals, in which the outer hair cells have been lost, suggesting that the preservation of the sensory afferents might be unique. Scale bar – 50 μm throughout.

**Fig. 6:**
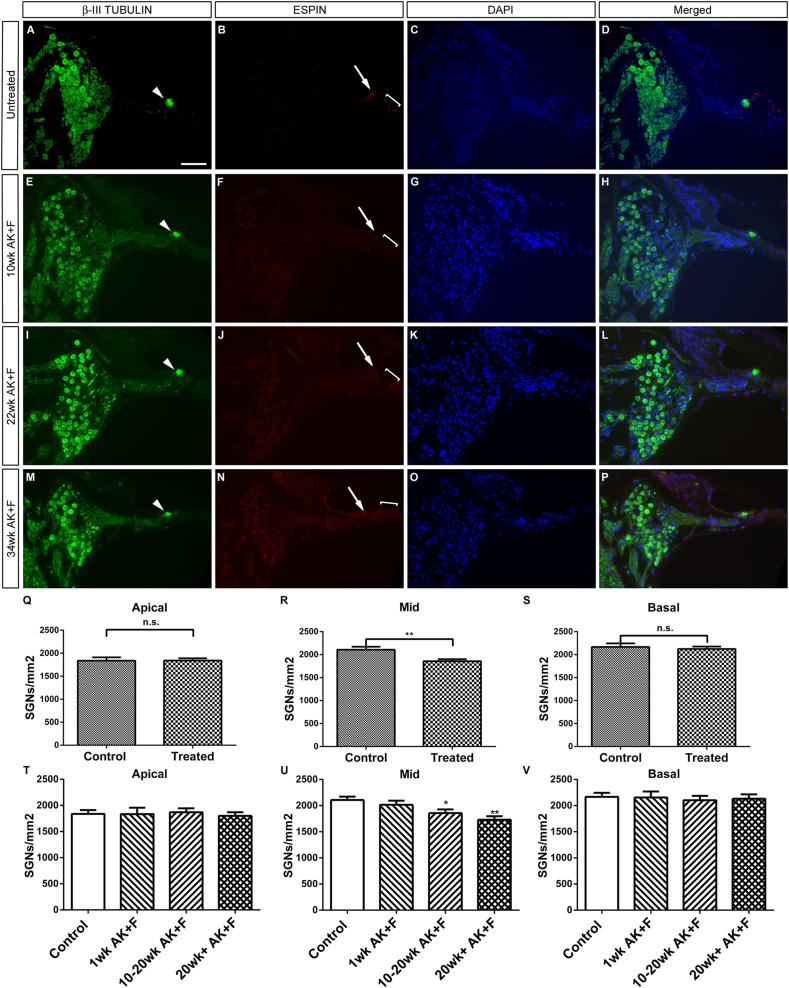
SGNs are preserved well after long-term kanamycin/furosemide-induced ototoxicity. SGNs residing in Rosenthal's canal were stained for the marker β-III TUBULIN and counted in untreated (A, D) and treated animals (E, H, I, L, M, P) at various time points post drug treatment. In all treated cases, immunostaining with the marker ESPIN demonstrates a loss of both inner (arrows – F, J, N) and outer hair cells (brackets – F, J, N) compared to the untreated situation (B); however in all cases, β-III TUBULIN staining suggests that innervation to the degenerating organ of Corti is maintained (arrowheads, E, I, M) as before (Fig. 3). There is no significant difference in the remaining number of SGNs at apical and basal levels of the cochlea, with a significant but mild loss in the mid-turn. (Q–S; **p < 0.05). A breakdown of the SGN counts between different groups at the different time points (T–V) demonstrates that the extent of SGN loss in the mid-turn occurs at 10–20wk post-treatment, becoming slightly worse at 20wk+ (U). Mean and SEM are plotted. Representative images for mid-apical turns are shown for each condition, with DAPI used as a nuclear counterstain. Scale bar – 100 μm, panels A–P.

**Fig. 7:**
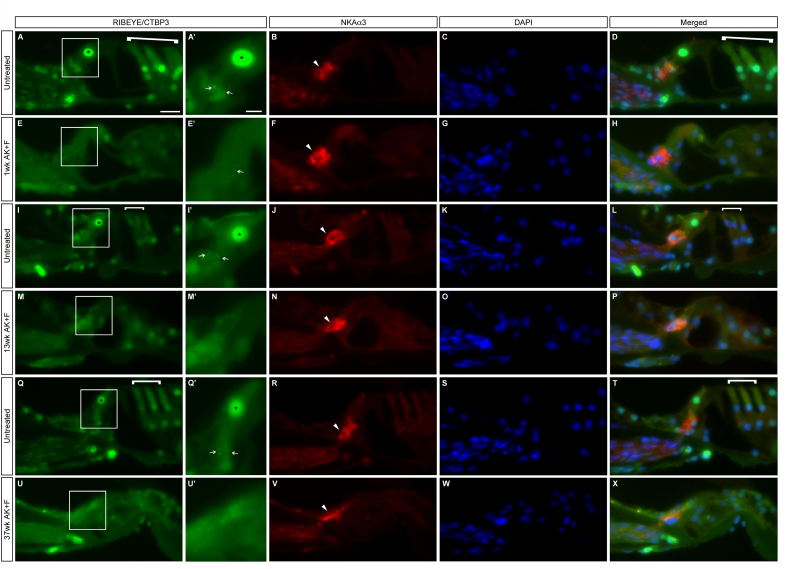
Sensory afferent neural processes remain into the organ of Corti in the absence of inner hair cells. Untreated (A–D, I–L, Q–T) and kanamycin/furosemide treated animals at different time points (1wk, E–H; 13wk M–P; 37wk U–X) were co-stained for the presence of the presynaptic ribbon component RIBEYE/CtBP3 and the sensory afferent marker Na, K-ATPase α3 subunit (NKAα3). In untreated samples, there is clear localisation of the RIBEYE protein at the base of the IHCs (arrows, A’, I’, Q’; apical, basal and mid turns respectively). This staining is mostly lost 1wk post-treatment (arrow, E’) and has completely gone at later stages (13wk, M’; 37wk, U’). There is a concomitant loss in treated samples of the nuclear localised RIBEYE protein which is seen in the IHCs of untreated samples (asterisks, A, A’; I, I’; M, M’), indicating a loss of these cells. The OHCs, indicated by brackets in A, D, I, L, Q, S, are also lost in the treated conditions. The SGN marker NKAα3 is maintained in all samples regardless of time post-treatment (arrowheads B, F, J, N, R, V), further confirming the residual ‘innervation’ of the organ of Corti months after the loss of the IHCs. Panels A’, E’, I’,M’, Q’ and U’ are magnified insets of the boxed regions in A, E, I, M, Q and U. Scale bars – 50 μm throughout panels A–X, while 10 μm in panels A’, E’, I’, M’, Q’, U’.

The inner and outer hair cells degenerate in response to the ototoxic insult (Figs. 3 and 4, S1, S2). However, it would appear that substantial innervation is maintained in the organ of Corti (Figs. 3, 4, 6 and 7, S1, S2). It has been suggested by previous workers (Stankovic et al., 2004; Sugawara et al., 2005; Zilberstein et al., 2012) that spiral ganglion neurons may still be receiving trophic support from surviving support cells in the organ of Corti – this may well indeed be the case, but interestingly the ‘knot’ of distal processes appears to be maintained even in cases where the entire organ appears to have been replaced by characteristic ‘damage’ epithelium (Fig. 3I, L).

### 3.7 Most spiral ganglion neurons are preserved after hair cell death

To see how this gerbil model of ototoxicity compares with other mammals, an investigation into spiral ganglion neuron density was carried out at varying time points after treatment. It was found that there is little loss of spiral ganglion neuron cell bodies in Rosenthal's canal, even up to 8 months post-treatment, in animals where hearing loss had been confirmed by the increase in click ABR threshold one week after the kanamycin/furosemide treatment (Fig. 6). Representative sections were assayed from the apical, mid and basal turns of the cochlea – in all cases, loss of hair cells was confirmed by the absence of co-labelling with the hair-cell specific markers ESPIN (Fig. 6) or MYOSIN7A (data not shown). As mentioned above, the axons innervating the organ of Corti remain in situ in the absence of the hair cells (denoted by white arrowheads in Fig. 6A, E, I, M) and do not die back. Average spiral ganglion cell numbers per unit area (mm^2^) were calculated in untreated, control animals (n = 5) and in cohorts of 1wk (n = 3); 10–20wk (n = 5) and 20wk+ (n = 4) post-treatment – see Tables 3 and 4 and Fig. 6. Cell numbers in the untreated condition were concordant with previously published data for gerbils of a similar age (Chen et al., 2012). A preliminary analysis of the data (Table 3, Fig. 6Q–S) shows that average SGN numbers are not reduced in the treated animals in the apical and basal turns, but that there is a mild reduction in cell number in the mid turn of the cochlea – the mean number of SGNs is reduced to an average of 1857/mm^2^ compared to 2106/mm^2^ in the control condition, a loss of approx. 12%. However, this is a minor loss compared to the large scale and rapid degeneration found in the guinea pig, for example (Bae et al., 2008) – an examination of the fluorescent micrographs gave a visual confirmation that there still remained a considerable number of SGNs, even at 34wk post-treatment (compare panels A, D with M,P, Fig. 6). Further analysis of the average SGN numbers remaining at particular timepoints clarified this loss (Table 4, Fig. 6T–V), confirming that it was indeed restricted to the mid turn of the cochlea and moreover, only occurred a substantial time after the ototoxic insult. – at 10–20wk after treatment, the loss was seen to be around 12% compared to the untreated situation; this figure only increased to approx. 18% at 20wk and beyond. A deeper analysis of individual animals showed that the reduction in SGN number was found in only two of the nine later cases studied, being present in one case in each cohort, despite all individuals having complete hair cell loss. As such, this may represent a minor variation between individuals in a population, similar to the variation in SGN loss observed after aminoglycoside-induced ototoxicity in the human (Huizing et al., 1987; Sone et al., 1998).

### 3.8 The remaining neurons are sensory afferents

To confirm the identity of the remaining ‘knot’ of neural processes at the organ of Corti, sections of treated cochleae at a range of timepoints post-kanamycin/furosemide treatment were stained for the presence of the sensory afferent marker, Na^+^ K^+^-ATPase α3 subunit (NKAα3), which is known to be expressed by Type I SGNs, particularly where these neurons ordinarily contact the hair cells (McGuirt et al., 1994; McLean et al., 2009). The remaining neurons were strongly immunoreactive for this marker (Fig. 7), at 1wk (Fig. 7F, H), 13wk (Fig. 7N, P) and 37wk post-treatment (Fig. 7V, X) implying that they are maintaining their sensory afferent identity despite the absence of the hair cells. The loss of the hair cells was also confirmed by the loss of RIBEYE/CtBP3 staining at the presynaptic ribbon of the IHC (Khimich et al., 2005) – in untreated samples (Fig. 7A, A’; I, I’; Q, Q’), there were discrete puncta of RIBEYE/CtBP3 localisation at the basal pole of the IHCs; this was mostly lost after kanamycin/furosemide treatment (Fig. 7E, E’; M, M’; U, U’). There was also a loss of the nuclear RIBEYE/CtBP3 staining (Knirsch et al., 2007), when the treated and untreated conditions are compared (asterisks, Fig. 7A, A’; I, I’; Q, Q’), which again illustrates the loss of the IHCs in treated animals. Interestingly, a smaller number of positive puncta were present a week after treatment (arrow, Fig. 7E’) but when the overall cell morphology and ABR decrement were taken into account (Fig. 7E’), this probably represented an IHC undergoing degenerative processes. A similar pattern was seen at all three axial levels of the cochlea – apical (Fig. 7A–H), mid (Fig. 7Q–X) and basal (Fig. 7I–P). Comparable results were obtained when samples were co-labelled for NKAα3 and the sensory afferent marker PARVALBUMIN (Fig. S2) – the latter is a marker for both neurons and inner hair cells. There was a stark loss of PARVALBUMIN staining from the IHCs of treated animals at 1wk (Fig. S2F), 13wk (Fig. S2N) and 37wk (Fig. S2V) compared with untreated controls (Fig. S2B, J and R); this loss was equivalent at the apical (Fig. S2A–H), mid (Fig. S2Q–X) and basal (Fig. S2I–P) levels of the cochlea. However, even in the absence of these cells, the knot of innervation by the sensory afferents remained as before (arrowheads, Fig. S2).

### 3.9 Myelination

**Fig. 8:**
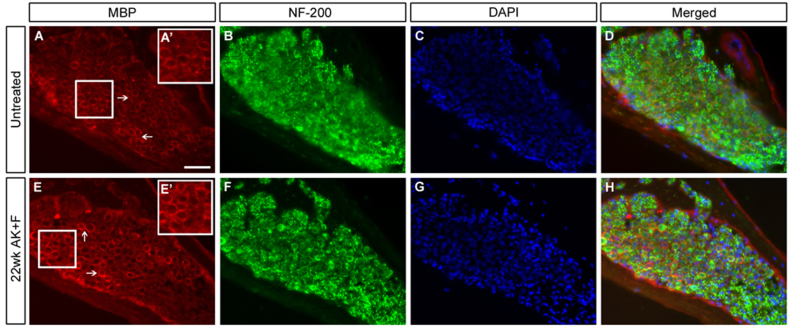
SGNs do not demyelinate in the long-term after kanamycin/furosemide treatment. Untreated (A–D) and a 22wk post-treatment (E–H) sample were examined for the expression of myelin basic protein (MBP, A, D, E, H) in Rosenthal's canal of the basal turn of the cochlea. Arrows (A, E) point to MBP staining ensheathing the SGNs. Inset boxes in A’, E’ represent 1.5× digitally magnified images of the corresponding boxed regions in A and E, respectively to show detail of the MBP sheath. There is no difference in expression apparent in the treated vs. the untreated condition. Counterstaining with neurofilament-200 (NF-200; B, F) showed the presence of nerve fibres in both cases; DAPI is used to counterstain the cell nuclei (C, D, G, H). Scale bar – 50 μm throughout.

A key marker of SGN degeneration after aminoglycoside treatment has been the loss of the myelin sheath coating the cell bodies and their processes, which has been shown for instance in the guinea pig (Koitchev et al., 1982) and the cat (Leake and Hradek, 1988) Examination of the myelination profile of the SGNs after long-term damage (Fig. 8) shows that the expression of myelin basic protein (MBP) remains indistinguishable from the untreated condition, even in an animal showing mild SGN loss. This suggests that the SGN profile is stable – the cells remaining at late stages after hair cell loss are not undergoing degenerative changes such as demyelination (Hurley et al., 2007; Ylikoski et al., 1974).

## 4 Discussion

### 4.1 The gerbil as a ‘one-shot’ model for ototoxicity

Here we present evidence that the Mongolian gerbil is a reliable model for ototoxic hearing loss in response to combinatorial treatment with kanamycin and furosemide and that a single dose regimen is adequate to induce rapid and profound hearing loss which does not improve over time. This means that this protocol can be used in conjunction with prior and subsequent surgical interventions, for instance in combination with the application of ouabain to the round window to create a model of auditory neuropathy; or with the insertion of an electrode to produce a cochlear implantation model. The efficiency of the ‘one-dose’ approach reduces the risks associated with chronic aminoglycoside treatment (for examples, see (Murillo-Cuesta et al., 2010)) and thus is preferable from an animal welfare, ‘3Rs’ perspective.

### 4.2 Gentamicin has little ototoxic effect in this model

As has been seen with other models such as the mouse (Murillo-Cuesta et al., 2010), these animals display differential sensitivities to different aminoglycosides, in that we do not see a substantial raising of the hearing threshold in response to gentamicin (Fig. 1), contrasting with the results seen by Sheppard et al. (2004). The results here do not necessarily conflict with the existing data – it may be that persistent and repeated application of the drug is required. When examining previous gentamicin usage in the gerbil, a shift in auditory threshold has been shown to occur after 4 weeks of daily, systemic injections (Chen et al., 1997), and hair cell loss has been demonstrated as being more substantial after five, daily transtympanic injections (Wanamaker et al., 1998), so whilst being effective, these methods lack the efficiency that was sought in developing this protocol. The fact that gentamicin does not impair the electromotile response of isolated gerbil outer hair cells is in line with the idea that hair cells are less sensitive to gentamicin damage in this species (Wang et al., 2007). In addition, Suryadevara et al. (2009) saw only a moderate reduction in cochlear hair cells (49% of IHCs, 34% of OHCs) two weeks after gentamicin was applied directly to the round window on a fibrin clot to facilitate slow release of the drug. In the light of this information combined with our own observations, we did not further pursue the use of gentamicin as an ototoxic agent in our model.

### 4.3 Use of gelatine sponge as a delivery vehicle

Gelatine sponge (‘Gelfoam’), has been widely used as a method of sustained, slow drug release and has been used clinically in the inner ear to ameliorate the symptoms of Meniere's disease (Silverstein et al., 1999). A substantial loss of cochlear hair cells was found two weeks after a gentamicin-soaked Gelfoam block was placed in apposition to the round window membrane (Sheppard et al., 2004). However, our functional data (Fig. 1) showed no effect of this when this experimental method was employed in our system. It may be however, that the polar nature of the gentamicin in aqueous solution may hinder its transit across the round window membrane; alternatively, the drug may become concentrated in the basal turn, affecting only high frequency hearing. We propose the former to be a more likely hypothesis, since there is no preferential loss of high frequency hearing in animals treated topically with gentamicin as assayed by pure tone-induced ABR. It could be argued, however, that the loss induced occurs at frequencies above the scope of our testing equipment but the histology of the treated cochleae shows that the organ of Corti remains intact throughout the basal turn and hook regions, suggesting that there is no build-up of the drug in these areas. Indeed, when kanamycin and furosemide were jointly applied on a gelatine sponge, a considerable shift in click ABR threshold was observed (40–50 dB increase; data not shown) after 2 weeks, and tone ABRs were lost across the frequency spectrum tested (6–38 kHz) in the treated ear, showing that these drugs could pass through the round window membrane and were evenly distributed throughout the endolymph. Interestingly, no loss of hearing was found in the untreated, control ear, implying that there is little, if any, meaningful diffusion of these drugs to the inner ear fluids of the contra-lateral side.

### 4.4 Potentiation of aminoglycoside ototoxicity with a loop diuretic

The use of an aminoglycoside antibiotic in isolation may be effective in some systems for inducing ototoxic damage, but this often requires a prolonged dosage regime, with associated side effects such as systemic toxicity and high mortality alongside what is frequently a comparatively mild hearing loss (see (Murillo-Cuesta et al., 2010; Poirrier et al., 2010) and references within for a more comprehensive treatment of this issue). Consequently, the experimental approach being pursued was altered to include a co-application of a loop diuretic, drugs which are well known to potentiate the effect of the antibiotics. For instance, a single systemic dose of kanamycin in the guinea pig had little effect on the cochlea until animals were also treated with ethacrynic acid, wherein complete destruction of the hair cells was observed (Brummett, 1981). We show that a single, systemic dose of kanamycin coupled with furosemide is an efficient method of generating substantial loss in hearing function. Interestingly, there is little to no effect on hearing when bumetanide is used as a potentiating agent, even when the drugs are applied topically to the round window. This was somewhat surprising, given that the pharmacology of these loop diuretics implies the activity of bumetanide to be many times greater than furosemide (Brummett et al., 1981), but may well reflect species-specific sensitivities – the guinea pig, for example, is more sensitive to ethacrynic acid than furosemide with respect to aminoglycoside-induced hair cell loss. We have also gathered preliminary data suggesting that kanamycin/furosemide is also effective when applied topically to the round window on a gelatin sponge, thus allowing for models of unilateral deafness to be developed.

We propose that all the hearing loss in evidence in our system arises as a direct result of the drug administration protocol, even in animals undergoing relatively ‘long-term’ treatment. Age-related hearing loss is known to occur in the Mongolian gerbil (Henry et al., 1980; Mills et al., 1990), however the functional loss we describe here arises rapidly and does not continue to decline over time, implying that there is no age-related component at play in our system – although it could be argued that age-related loss occurs at a time-point beyond the duration of the experiments conducted here, this is beyond the scope of the work discussed in this manuscript. Additionally, there is no evidence of cholesteatoma in our colony, as has been seen by others (Chole et al., 1981), giving further weight to our proposal that the deafness we observe is entirely due to the kanamycin/furosemide treatment paradigm.

### 4.5 Different species respond in different ways to aminoglycosides

There is a wide-range of literature on the use of animal models for aminoglycoside-induced ototoxicity, surveying the use of different compounds in conjunction with different diuretics. The data within the literature is often contradictory – for instance, Poirrier et al. saw no effect on ABR or hair cell loss upon sustained treatment with kanamycin in mice (Poirrier et al., 2010) whereas a high-frequency hearing loss, consistent with concentration of the kanamycin within the basal turn, was seen in mice by Hirose and Sato; moreover, Taylor et al. reported a complete abrogation of OHCs 48 h after the application of kanamycin and bumetanide (Hirose et al., 2011; Taylor et al., 2008).

Nevertheless, it is generally accepted that mice are less susceptible to aminoglycoside damage than for instance, guinea pigs – the former are renowned for requiring a high and frequent (e.g. every 12 h for 15 days) dosing regimen to induce a highly variable and often milder ototoxic lesion (see Poirrier et al., 2010 for a comprehensive covering of this material). In contrast, the comparative efficiency of deafening in the guinea pig has been well established over the past four decades, in a procedure pioneered by the Brummett group and continued by many others, for instance van Loon et al (2013) (Brummett et al., 1975; van Loon et al., 2013). This has led to the guinea pig being proposed as a better model for ototoxic hearing loss in the human, where aminoglycosides have historically been found to have unfortunate side-effects on hearing function (Matz et al., 1965).

It remains open to conjecture as to the differential sensitivity of our experimental gerbils to gentamicin as compared to those treated by Sheppard et al. (2004), in which a near complete destruction of hair cells was achieved. Laboratory gerbils are maintained in relatively small colonies, so it may be that different groups of workers have inadvertently selected for differential drug sensitivities; alternatively, the age of onset of the treatment may be a contributing factor – gerbils are typically 12 weeks and above when entering our experimental schedule, whereas an initiating age of 5–8 weeks was used by the previous authors.

The primary aim of this study was to investigate the behaviour of the gerbil sensorineural hearing system when challenged with aminoglycosides – the data here suggests that hair cell loss and hearing function is not particularly susceptible to gentamicin or kanamycin in isolation.

### 4.6 The preservation of spiral ganglion neurons is unusual in commonly used small laboratory mammals

A notable result found in here is the preservation of both type I and type II spiral ganglion neurons long after the ototoxic lesion has occurred – animals were followed for over 8 months and no appreciable loss of neurons was noted, with cell counts at the base and the apex of the cochlea being equivalent to those in untreated animals and cell numbers in the mid-turn being mildly reduced in a couple of animals. This is relatively unusual amongst rodents and other small mammalian hair cell loss models – both in the chinchilla and the guinea pig, secondary degeneration of the SGNs occurs as a rapid consequence of the ototoxic lesion (Kong et al., 2010; McFadden et al., 2004; Versnel et al., 2007; Webster et al., 1981). However, the situation described here is very similar to cases which have been described in human patients with hearing loss. In cases of long-term gentamicin ototoxicity in the human a bilateral loss of the organ of Corti was found in both ears, however, SGNs were found to be largely unaffected (Huizing and de Groot, 1987). Similarly, in a review of the histology of profoundly deaf patients (Nadol, 1997), the samples coming from those with aminoglycoside-induced toxicity were found to have the highest residual SGN counts compared with congenital hearing loss. Furthermore, histological analysis of a temporal bone repository showed that loss of hair cells was not accompanied by a proportional loss of spiral ganglion neurons in the equivalent, tonotopical regions (Linthicum and Fayad, 2009). This is in agreement with our findings for the gerbil.

The continued presence of nerve fibres within the organ of Corti months after the hair cells have been lost is an unusual feature in our model and suggests that the remaining support cells may be maintaining their identity and their trophic actions on the peripheral nerve terminals. The first sign of retrograde degeneration of the SGNs has been suggested to be the ‘dying back’ of these fibres (Dodson and Mohuiddin, 2000) (and references within) – in the gerbil model we describe here, the peripheral fibres of the Type I SGNs still form a ‘knot-like’ structure beneath the position of the destroyed inner hair cells and no retrograde degeneration is observed. Nor is there a reduction in the myelination of these peripheral processes, as has been proposed to occur alongside the neural degeneration post-deafening in the rat, for example (Hurley et al., 2007). It has been long been proposed that the inner hair cells give trophic support to the SGNs – for example (Ylikoski et al., 1974) – and that the presence of remaining support cells has no bearing on SGN survival (Bichler et al., 1983). However, our data add weight to the proposal that the support cells are central to SGN survival (Schuknecht, 1953; Zilberstein et al., 2012), the key new finding here being that the peripheral neural processes in the organ of Corti are maintained even in the presence of so-called ‘flattened’ epithelium.

### 4.7 Support cell morphology

We observe that in some cases, the organ of Corti remains intact, lacking hair cells, for several months after the drug treatment, whereas in others, the transition to a flat, ‘damage’ epithelium appears to be ongoing, with both of these phenomena having been reported previously (Oesterle et al., 2009; Sugawara et al., 2005; Taylor et al., 2012). This raises interesting questions regarding the identity and differentiation state of these cells. Although clearly not ‘normal’ in appearance, the perdurance of their characteristic support cell markers coupled with the continued presence of SGN innervation at the site of the IHCs implies that they may still be capable of supplying trophic support to the system. When hair cells die in mammals, a permanent scar of ‘flat’ epithelium eventually replaces the organ of Corti (reviewed in (Raphael et al., 2007)); debate remains in the literature as to whether the eventual loss of the support cells is a secondary consequence of hair cell loss, or results from primary entry of the aminoglycoside into these cells, which has been demonstrated in some models (Dai et al., 2006; Wang et al., 2009); indeed the catastrophic and rapid loss of the organ a week after treatment in the guinea pig has been proposed by Oesterle to result from such primary damage to the support cells (Oesterle, 2013). This scar formation of flattened epithelium is thought to allow a certain degree of repair to the damaged organ and prevent further insult to any remaining hair cells which would occur if the perilymph and endolymph were allowed to mix. The triggers for the transition from ‘damaged’ organ of Corti to ‘flattened’ epithelium are currently unknown (Oesterle, 2013) and the identity of cells bringing about the repair is under debate – are they support cells which de-differentiate from a columnar to a cuboidal epithelium, or are they a population of sulcus cells which migrate in to replace dead support cells? The long-term perdurance of support cell markers such as OCP2 and acetylated αTUBULIN described here underpins the former proposal and is supported by data from others, in which the ‘repaired organ of Corti’ has been described (Oesterle et al., 2009; Sugawara et al., 2005; Taylor et al., 2012).

We do see occasional remnants of hair cell marker expression – for instance, the trace of ESPIN seen in Fig. 3M. However, the morphology of these cells is abnormal, and may be ones that are undergoing phagocytosis by neighbouring support cells or circulating white blood cells to remove them from the damaged epithelium (Abrashkin et al., 2006; Hirose et al., 2005).

## 5 Conclusions

The ease with which ototoxic lesions may be induced in the gerbil, as shown here, adds further credence to the use of this animal as an experimental model for hearing research. Moreover, while cats and primates manifest a similar preservation of the auditory neurons for a long time post-insult, this is somewhat unique amongst commonly-used small laboratory animals, and reflects more accurately the human situation, where spiral ganglion neurons may remain for substantial lengths of time after hair cell loss. Given the larger costs and deeper ethical conundrums associated with experimentation in cats, dogs and primates, the gerbil offers a reasonable, complementary model system. We are combining this treatment with the selective ablation of spiral ganglion neurons using ouabain (Schmiedt et al., 2002), to generate a ‘double knock-down’ model. This should provide an ideal system to investigate cochlear implantation using an implantable stimulator (Millard and Shepherd, 2007), together with restoration of auditory nerve function by the engraftment of transplanted otic neural progenitors (Chen et al., 2012).

## Figures and Tables

**Table 1:** Measuring the ABR resulting from a range of individual tone frequencies shows a significant increase in auditory threshold across a broad frequency spectrum.

Frequency (kHz)	Average threshold before K/F (dB SPL)	Average threshold after K/F (dB SPL)	Δ Threshold (dB SPL)	Significant?
2	29	87	58	Yes****
4	26	85	59	Yes****
8	53	88	35	Yes****
16	58	82	24	Yes****
32	65	87	22	Yes****

**Table 2:** IHC and OHC counts from untreated and kanamycin/furosemide treated animals after 1 wk. ‘n’ refers to the number of cochleae counted per condition and come from 2 (untreated condition) or 3 (treated condition) different animals. There is a significant loss of hair cells at each turn of the cochlea.

	Apical			Mid			Basal		
	Cells/mm^2^	n	*p* < 0.05	Cells/mm^2^	n	*p* < 0.05	Cells/mm^2^	n	*p* < 0.05
Untreated IHCS	113.8	2		99.8	2		103.9	2	
Treated IHCs	0	5	Yes****	21.6	5	Yes*	19.7	3	Yes*
Untreated OHCs	457.8	2		393.3	2		402.5	2	
Treated OHCs	0	5	Yes****	1.8	5	Yes****	0	3	Yes***

**Table 3:** Analysis of mean SGN number at each level of the cochlea in treated animals (n = 12) and untreated controls (n = 5). A significant loss of SGNs is only seen in the mid-turn of the cochlea in treated animals. ‘n’ refers to the number of animals counted per condition.

	Apical			Mid			Basal		
	Cells/mm^2^	n	p < 0.05?	Cells/mm^2^	n	p < 0.05?	Cells/mm^2^	n	p < 0.05?
Untreated	1839	5		2106	5		2168	5	
Treated	1842	12	**No**	1857	12	Yes**	2124	12	**No**

**Table 4:** Analysis of mean SGN number at each level of the cochlea for each treated time point cohort. The significant variation in the mid-turn counts results from SGN losses at later stages, being evident at 10wk and more noticeable at 20wk post-treatment. ‘n’ refers to the number of animals counted per condition.

	Apical			Mid			Basal		
	Cells/mm^2^	n	p < 0.05?	Cells/mm^2^	n	p < 0.05?	Cells/mm^2^	n	p < 0.05?
Untreated	1839	5	n/a	2106	5	n/a	2168	5	n/a
1wk	1834	3	No	2016	3	No	2156	3	No
10–20wk	1870	5	No	1857	5	Yes*	2104	5	No
20wk+	1802	4	No	1730	4	Yes**	2132	4	No

## References

[bib1] Abrashkin K.A., Izumikawa M., Miyazawa T., Wang C.H., Crumling M.A., Swiderski D.L., Beyer L.A., Gong T.W., Raphael Y. (2006). The fate of outer hair cells after acoustic or ototoxic insults. Hear Res..

[bib2] Alam S.A., Ikeda K., Kawase T., Kikuchi T., Katori Y., Watanabe K., Takasaka T. (1998). Acute effects of combined administration of kanamycin and furosemide on the stria vascularis studied by distortion product otoacoustic emission and transmission electron microscopy. Tohoku J. Exp. Med..

[bib3] Aran J.M., Darrouzet J. (1975). Observation of click-evoked compound VIII nerve responses before, during, and over seven months after kanamycin treatment in the guinea pig. Acta Otolaryngol..

[bib4] Astbury P.J., Read N.G. (1982). Kanamycin induced ototoxicity in the laboratory rat. A comparative morphological and audiometric study. Arch. Toxicol..

[bib5] Bae W.Y., Kim L.S., Hur D.Y., Jeong S.W., Kim J.R. (2008). Secondary apoptosis of spiral ganglion cells induced by aminoglycoside: Fas-Fas ligand signaling pathway. Laryngoscope.

[bib6] Bichler E., Spoendlin H., Rauchegger H. (1983). Degeneration of cochlear neurons after amikacin intoxication in the rat. Arch. Otorhinolaryngol..

[bib7] Boettcher F.A., Emery M. (2006). Auditory evoked-potential correlates of decrement detection. Hear Res..

[bib8] Brown C.B., Ogg C.S., Cameron J.S., Bewick M. (1974). High dose frusemide in acute reversible intrinsic renal failure. A preliminary communication. Scott Med. J..

[bib9] Brummett R.E. (1981). Effects of antibiotic-diuretic interactions in the guinea pig model of ototoxicity. Rev. Infect. Dis..

[bib10] Brummett R.E., Bendrick T., Himes D. (1981). Comparative ototoxicity of bumetanide and furosemide when used in combination with kanamycin. J. Clin. Pharmacol..

[bib11] Brummett R.E., Traynor J., Brown R., Himes D. (1975). Cochlear damage resulting from kanamycin and furosemide. Acta Otolaryngol..

[bib12] Burkard R., Boettcher F., Voigt H., Mills J. (1993). Comments on “Stimulus dependencies of the gerbil brain-stem auditory-evoked response (BAER). I: effects of click level, rate and polarity” [J. Acoust. Soc. Am. 85, 2514–2525 (1989)]. J. Acoust. Soc. Am..

[bib13] Chen T.J., Chen S.S., Lin C.H., Hsieh Y.L. (1997). Synergestic effects of noise and aminoglycoside antibiotic (gentamicin) on auditory function in the gerbil. Kaohsiung J. Med. Sci..

[bib14] Chen W., Jongkamonwiwat N., Abbas L., Eshtan S.J., Johnson S.L., Kuhn S., Milo M., Thurlow J.K., Andrews P.W., Marcotti W., Moore H.D., Rivolta M.N. (2012). Restoration of auditory evoked responses by human ES-cell-derived otic progenitors. Nature.

[bib15] Chole R.A., Henry K.R., McGinn M.D. (1981). Cholesteatoma: spontaneous occurrence in the Mongolian gerbil Meriones unguiculatis. Am. J. Otol..

[bib16] Dai C.F., Mangiardi D., Cotanche D.A., Steyger P.S. (2006). Uptake of fluorescent gentamicin by vertebrate sensory cells in vivo. Hear Res..

[bib17] Dodson H.C., Mohuiddin A. (2000). Response of spiral ganglion neurones to cochlear hair cell destruction in the guinea pig. J. Neurocytol..

[bib18] Ghorayer B., Sarwat A., Linthicum F.H. (1980). Viable spiral ganglion cells in congenital and acquired profound hearing loss. J. Laryngol. Otol..

[bib19] Hallworth R., Luduena R.F. (2000). Differential expression of beta tubulin isotypes in the adult gerbil cochlea. Hear Res..

[bib20] Hellier W.P., Wagstaff S.A., O'Leary S.J., Shepherd R.K. (2002). Functional and morphological response of the stria vascularis following a sensorineural hearing loss. Hear Res..

[bib21] Henry K.R., McGinn M.D., Chole R.A. (1980). Age-related auditory loss in the Mongolian gerbil. Arch. Otorhinolaryngol..

[bib22] Henzl M.T., Thalmann I., Larson J.D., Ignatova E.G., Thalmann R. (2004). The cochlear F-box protein OCP1 associates with OCP2 and connexin 26. Hear Res..

[bib23] Hirose K., Sato E. (2011). Comparative analysis of combination kanamycin-furosemide versus kanamycin alone in the mouse cochlea. Hear Res..

[bib24] Hirose K., Discolo C.M., Keasler J.R., Ransohoff R. (2005). Mononuclear phagocytes migrate into the murine cochlea after acoustic trauma. J. Comp. Neurol..

[bib25] Huizing E.H., de Groot J.C. (1987). Human cochlear pathology in aminoglycoside ototoxicity – a review. Acta Otolaryngol. Suppl..

[bib26] Hurley P.A., Crook J.M., Shepherd R.K. (2007). Schwann cells revert to non-myelinating phenotypes in the deafened rat cochlea. Eur. J. Neurosci..

[bib27] Khimich D., Nouvian R., Pujol R., Tom Dieck S., Egner A., Gundelfinger E.D., Moser T. (2005). Hair cell synaptic ribbons are essential for synchronous auditory signalling. Nature.

[bib28] Knirsch M., Brandt N., Braig C., Kuhn S., Hirt B., Munkner S., Knipper M., Engel J. (2007). Persistence of Ca(v)1.3 Ca^2+^ channels in mature outer hair cells supports outer hair cell afferent signaling. J. Neurosci..

[bib29] Koitchev K., Guilhaume A., Cazals Y., Aran J.M. (1982). Spiral ganglion changes after massive aminoglycoside treatment in the guinea pig. Counts and ultrastructure. Acta Otolaryngol..

[bib30] Kong W.J., Yin Z.D., Fan G.R., Li D., Huang X. (2010). Time sequence of auditory nerve and spiral ganglion cell degeneration following chronic kanamycin-induced deafness in the guinea pig. Brain Res..

[bib31] Lang H., Schulte B.A., Schmiedt R.A. (2005). Ouabain induces apoptotic cell death in type I spiral ganglion neurons, but not type II neurons. J. Assoc. Res. Otolaryngol..

[bib32] Leake P.A., Hradek G.T. (1988). Cochlear pathology of long term neomycin induced deafness in cats. Hear Res..

[bib33] Linthicum F.H., Fayad J.N. (2009). Spiral ganglion cell loss is unrelated to segmental cochlear sensory system degeneration in humans. Otol. Neurotol..

[bib34] MacDonald G.H., Rubel E.W. (2008). Three-dimensional imaging of the intact mouse cochlea by fluorescent laser scanning confocal microscopy. Hear Res..

[bib35] Mathog R.H., Klein W.J. (1969). Ototoxicity of ethacrynic acid and aminoglycoside antibiotics in uremia. N. Engl. J. Med..

[bib36] Matz G.J., Wallace T.H., Ward P.H. (1965). The ototoxicity of kanamycin. A comparative histopathological study. Laryngoscope.

[bib37] May B.J., Prosen C.A., Weiss D., Vetter D. (2002). Behavioral investigation of some possible effects of the central olivocochlear pathways in transgenic mice. Hear. Res..

[bib38] McFadden S.L., Ding D., Jiang H., Salvi R.J. (2004). Time course of efferent fiber and spiral ganglion cell degeneration following complete hair cell loss in the chinchilla. Brain Res..

[bib39] McGuirt J.P., Schulte B.A. (1994). Distribution of immunoreactive alpha- and beta-subunit isoforms of Na,K-ATPase in the gerbil inner ear. J. Histochem. Cytochem..

[bib40] McLean W.J., Smith K.A., Glowatzki E., Pyott S.J. (2009). Distribution of the Na,K-ATPase alpha subunit in the rat spiral ganglion and organ of corti. J. Assoc. Res. Otolaryngol..

[bib41] Millard R.E., Shepherd R.K. (2007). A fully implantable stimulator for use in small laboratory animals. J. Neurosci. Methods.

[bib42] Mills J.H., Schmiedt R.A., Kulish L.F. (1990). Age-related changes in auditory potentials of Mongolian gerbil. Hear Res..

[bib43] Murillo-Cuesta S., Contreras J., Cediel R., Varela-Nieto I. (2010). Comparison of different aminoglycoside antibiotic treatments to refine ototoxicity studies in adult mice. Lab. Anim..

[bib44] Nadol J.B. (1997). Patterns of neural degeneration in the human cochlea and auditory nerve: implications for cochlear implantation. Otolaryngol. Head. Neck Surg..

[bib45] Oesterle E.C. (2013). Changes in the adult vertebrate auditory sensory epithelium after trauma. Hear Res..

[bib46] Oesterle E.C., Campbell S. (2009). Supporting cell characteristics in long-deafened aged mouse ears. J. Assoc. Res. Otolaryngol..

[bib47] Otto G., Jürgen S. (2012). The Mongolian Gerbil as a Model for the Analysis of Peripheral and Central Age-dependent Hearing Loss.

[bib48] Poirrier A.L., Van den Ackerveken P., Kim T.S., Vandenbosch R., Nguyen L., Lefebvre P.P., Malgrange B. (2010). Ototoxic drugs: difference in sensitivity between mice and guinea pigs. Toxicol. Lett..

[bib49] Polgar R., Collison T., Slepecky N.B., Wanamaker H.H. (2001). Anatomic and morphometric changes to gerbil posterior cristas following transtympanic administration of gentamicin and streptomycin. J. Assoc. Res. Otolaryngol..

[bib50] Raphael Y., Kim Y.H., Osumi Y., Izumikawa M. (2007). Non-sensory cells in the deafened organ of Corti: approaches for repair. Int. J. Dev. Biol..

[bib51] Saha S., Slepecky N.B. (2000). Age-related changes in microtubules in the guinea pig organ of Corti. Tubulin isoform shifts with increasing age suggest changes in micromechanical properties of the sensory epithelium. Cell. Tissue Res..

[bib52] Schmiedt R.A., Okamura H.O., Lang H., Schulte B.A. (2002). Ouabain application to the round window of the gerbil cochlea: a model of auditory neuropathy and apoptosis. J. Assoc. Res. Otolaryngol..

[bib53] Schuknecht H.F. (1953). Lesions of the organ of Corti. Trans. Am. Acad. Ophthalmol. Otolaryngol..

[bib54] Sheppard W.M., Wanamaker H.H., Pack A., Yamamoto S., Slepecky N. (2004). Direct round window application of gentamicin with varying delivery vehicles: a comparison of ototoxicity. Otolaryngol. Head. Neck Surg..

[bib55] Silverstein H., Arruda J., Rosenberg S.I., Deems D., Hester T.O. (1999). Direct round window membrane application of gentamicin in the treatment of Meniere's disease. Otolaryngol. Head. Neck Surg..

[bib56] Sone M., Schachern P.A., Paparella M.M. (1998). Loss of spiral ganglion cells as primary manifestation of aminoglycoside ototoxicity. Hear Res..

[bib57] Stankovic K., Rio C., Xia A., Sugawara M., Adams J.C., Liberman M.C., Corfas G. (2004). Survival of adult spiral ganglion neurons requires erbB receptor signaling in the inner ear. J. Neurosci..

[bib58] Sugawara M., Corfas G., Liberman M.C. (2005). Influence of supporting cells on neuronal degeneration after hair cell loss. J. Assoc. Res. Otolaryngol..

[bib59] Suryadevara A.C., Wanamaker H.H., Pack A. (2009). The effects of sound conditioning on gentamicin-induced vestibulocochlear toxicity in gerbils. Laryngoscope.

[bib60] Tannenbaum J., Slepecky N.B. (1997). Localization of microtubules containing posttranslationally modified tubulin in cochlear epithelial cells during development. Cell. Motil. Cytoskelet..

[bib61] Taylor R.R., Nevill G., Forge A. (2008). Rapid hair cell loss: a mouse model for cochlear lesions. J. Assoc. Res. Otolaryngol..

[bib62] Taylor R.R., Jagger D.J., Forge A. (2012). Defining the cellular environment in the organ of Corti following extensive hair cell loss: a basis for future sensory cell replacement in the Cochlea. PLoS One.

[bib63] van Loon M.C., Ramekers D., Agterberg M.J., de Groot J.C., Grolman W., Klis S.F., Versnel H. (2013). Spiral ganglion cell morphology in guinea pigs after deafening and neurotrophic treatment. Hear Res..

[bib64] Versnel H., Agterberg M.J., de Groot J.C., Smoorenburg G.F., Klis S.F. (2007). Time course of cochlear electrophysiology and morphology after combined administration of kanamycin and furosemide. Hear Res..

[bib65] Wanamaker H.H., Slepecky N.B., Cefaratti L.K., Ogata Y. (1999). Comparison of vestibular and cochlear ototoxicity from transtympanic streptomycin administration. Am. J. Otol..

[bib66] Wanamaker H.H., Gruenwald L., Damm K.J., Ogata Y., Slepecky N. (1998). Dose-related vestibular and cochlear effects of transtympanic gentamicin. Am. J. Otol..

[bib67] Wang Q., Steyger P.S. (2009). Trafficking of systemic fluorescent gentamicin into the cochlea and hair cells. J. Assoc. Res. Otolaryngol..

[bib68] Wang X., Jia S., Currall B., Yang S., He D.Z. (2007). Streptomycin and gentamicin have no immediate effect on outer hair cell electromotility. Hear Res..

[bib69] Webster M., Webster D.B. (1981). Spiral ganglion neuron loss following organ of Corti loss: a quantitative study. Brain Res..

[bib70] Yang T.H., Liu S.H., Young Y.H. (2010). A novel inner ear monitoring system for evaluating ototoxicity of gentamicin eardrops in guinea pigs. Laryngoscope.

[bib71] Ylikoski J., Wersall J., Bjorkroth B. (1974). Degeneration of neural elements in the cochlea of the guinea-pig after damage to the organ of corti by ototoxic antibiotics. Acta Otolaryngol. Suppl..

[bib72] Zilberstein Y., Liberman M.C., Corfas G. (2012). Inner hair cells are not required for survival of spiral ganglion neurons in the adult cochlea. J. Neurosci..

